# Babesiosis in Fairfield County, Connecticut

**DOI:** 10.3201/eid1003.030561

**Published:** 2004-03

**Authors:** John F. Anderson, Louis A. Magnarelli

**Affiliations:** * Connecticut Agricultural Experiment Station, New Haven, Connecticut, USA

**To the Editor**: Human babesiosis, caused by *Babesia microti*, was initially described in the eastern United States in 1970 in a woman vacationing on Nantucket Island, Massachusetts (*1*). With few exceptions, almost all subsequent cases were recorded from islands in the northeastern United States and Cape Cod, Massachusetts (*2*), until this illness was diagnosed in 13 patients living in New London County in southeastern Connecticut (*3*,*4*). *B. microti* was isolated from white-footed mice, *Peromyscus leucopus*, captured from 1988 to 1990 in the yards of patients. Babesiosis also was diagnosed in persons living in Wisconsin (*5*) and in New Jersey (*6*) who acquired the organism locally. The number of cases of babesiosis reported by health departments on their Web sites and by personal communication in Massachusetts, Rhode Island, and New York State, was 330 from 1988 to 2002, 121 from 1994 to 2002, and 542 from 1986 to 2001, respectively. The number of cases reported by the New York City Health Department from 1991 to 2000 was 75.

From 1991 to 2000, babesiosis was diagnosed in 230 persons residing in New London County and adjacent Middlesex County, Connecticut (*7*). Fifty-three additional cases were reported in five other counties in Connecticut, but epidemiologic data did not indicate that these infections likely were acquired within Connecticut. We now note a new and distinct geographic focus by reporting the isolation of *B. microti* from rodents captured in the yards of two patients in whom babesiosis was diagnosed at Greenwich Hospital in 2002. These patients lived in Greenwich, Connecticut, which is located in Fairfield County in the extreme southwestern part of the state. Neither patient had traveled outside of the immediate area of Greenwich, Connecticut, before onset of illness. We also trapped rodents in the yards of two additional patients in whom babesiosis was diagnosed. These two patients had traveled to Rhode Island shortly before becoming ill. Patients became ill from June 23 to July 7, 2002, and none reported a tick bite.

Attempts to trap small mammals on the properties of the four patients were made on July 22, 23, and 29, 2002. Rodents were captured in Sherman box traps baited with peanut butter and apple. Approximately 0.3 mL of blood was drawn from the heart of each animal into a syringe coated with heparin or uncoated. Blood was kept on ice in the field and then returned to the laboratory. A 3- to 5-week-old male Syrian hamster was injected intraperitoneally with 0.1 mL of each blood sample.

Blood smears were obtained from a drop of blood taken from the tail of each hamster on weeks 3 to 6 after injection. Blood cells were stained with Giemsa and examined for *B. microti* at a magnification of 1,008X. Hamsters were considered uninfected when no parasites were found in 75 fields of stained erythrocytes.

*B. microti* was isolated from rodents captured at the residences of two of the patients who did not travel outside of the Greenwich area 6 weeks before onset of illness. Blood from two of three white-footed mice and from the two eastern chipmunks, *Tamias striatus*, captured in the yards of the patients, produced infections in injected hamsters. Infections did not develop in hamsters injected with blood from 10 white-footed mice captured at the residences of two patients who visited Wakefield and Charlestown, Rhode Island, shortly before becoming ill.

*B. microti* is prevalent in rodent populations in Greenwich, Connecticut, and causes human disease. Establishing evidence of *B. microti* in rodents and documenting this protozoan parasite as the cause of human disease in Greenwich are important. Relatively high populations of the vector tick, *Ixodes scapularis*, are present in Greenwich and nearby towns. In 2002, the health departments of Greenwich, Stamford, New Canaan, and Darien submitted 1,671 *I. scapularis* ticks removed from persons to the Connecticut Agricultural Experiment Station for identification and testing for *Borrelia burgdorferi*. Two hundred and thirty cases of Lyme disease were reported from these four towns in 2002 (Connecticut Department of Public Health, unpub. data). With such extensive human exposure to ticks and a relatively large number of Lyme disease cases in these four towns and elsewhere in Fairfield County, the number of cases of babesiosis is likely to increase appreciably in the future.

*B. microti* has been transmitted through blood transfusion in Connecticut (*8*). Blood collection agencies in southwestern Connecticut and adjacent Westchester County, New York, should be aware of the possibility that blood donors could be infected with this pathogen. Physicians should also be alert to the possibility that patients could be coinfected with the etiologic agents of Lyme disease or human granulocytic ehrlichiosis. Some patients in whom Lyme disease was diagnosed have been simultaneously infected with *B. microti* (*9*,*10*).
